# Rate-Responsive Cardiac Pacing: Technological Solutions and Their Applications

**DOI:** 10.3390/s23031427

**Published:** 2023-01-27

**Authors:** Ewa Świerżyńska, Artur Oręziak, Renata Główczyńska, Antonio Rossillo, Marcin Grabowski, Łukasz Szumowski, Francesco Caprioglio, Maciej Sterliński

**Affiliations:** 1 Department of Arrhythmia, The Cardinal Stefan Wyszynski National Institute of Cardiology, 04-628 Warsaw, Poland; 2 1st Department of Cardiology, Medical University of Warsaw, 02-097 Warsaw, Poland; 3 Department of Cardiology, San Bortolo Hospital, 36100 Vicenza, Italy

**Keywords:** cardiac pacemakers, rate response, chronotropic incompetence, physical exercise, physical capacity

## Abstract

Modern cardiac pacemakers are equipped with a function that allows the heart rate to adapt to the current needs of the patient in situations of increased demand related to exercise and stress ("rate-response" function). This function may be based on a variety of mechanisms, such as a built-in accelerometer responding to increased chest movement or algorithms sensing metabolic demand for oxygen, analysis of intrathoracic impedance, and analysis of the heart rhythm (Q-T interval). The latest technologies in the field of rate-response functionality relate to the use of an accelerometer in leadless endocavitary pacemakers; in these devices, the accelerometer enables mapping of the mechanical wave of the heart’s work cycle, enabling the pacemaker to correctly sense native impulses and stimulate the ventricles in synchrony with the cycles of atria and heart valves. Another modern system for synchronizing pacing rate with the patient’s real-time needs requires a closed-loop system that continuously monitors changes in the dynamics of heart contractions. This article discusses the technical details of various solutions for detecting and responding to situations related to increased oxygen demand (e.g., exercise or stress) in implantable pacemakers, and reviews the results of clinical trials regarding the use of these algorithms.

## 1. Introduction

Chronotropic incompetence (CI) refers to a heart’s inability to adapt (increase to reach 85% of the maximum age-predicted heart rate) its rate to improve cardiac output to meet metabolic demands during exercise and stress. In this disorder, an inadequate heart rate is generated relative to the needs of the organism, resulting in an insufficient blood supply, fatigue, shortness of breath, limited ability to exercise, and a reduced quality of life [1]. CI is an independent predictor of major adverse cardiovascular events and overall mortality [2]. The disorder is often associated with other dysfunctions of the cardiovascular system, including heart failure [3,4]. Implanted pacemakers have been successfully used to treat some of these dysfunctions [5]. Modern pacemakers are equipped with a number of functions and algorithms that adjust the basal rate of pacing to situations associated with increased demands of the body—this requires sensors for accelerometer-based measurements; measurements of minute ventilation; measurements of myocardial contractility; and the analysis of myocardial, transthoracic, and transvalvular impedances [6]. Some of these functions are based on processing or mapping an electrocardiography (ECG) signal, including analysis of the QT interval or processing of the vibration associated with valve closure into the mapping of the atrioventricular interval in ventricular pacing triggered by atrial sensed beats (VDD mode) with the use of a leadless pacemaker.

## 2. Chronotropic Incompetence

The sympathetic branch of the autonomic nervous system is responsible for accelerating the heart rate according to the current body state, among other functions. The primary neurotransmitter released by the sympathetic nervous system is norepinephrine. The autonomic system innervates the heart through the jugular ganglia and the cardiac plexus—the body’s natural pacemakers. The heart rate reflects the balance between the sympathetic and parasympathetic parts of the autonomic nervous system [7]. The sinoatrial node is responsible for generating and regulating the heart rate. In a healthy person, the sinoatrial node generates impulses that are then conducted through the fibers of the conduction system to the atrial musculature and to the atrioventricular node and the His–Purkinje system, ultimately causing excitation of the muscles of the right and left ventricles of the heart. Dysfunctions of the cardiac conduction system can lead to the development of numerous disorders of heart function, including heart failure. Some of these dysfunctions, such as atrioventricular conduction blocks (2nd and 3rd degree or complete block) and diseases of the sinus node (tachycardia–bradycardia syndrome, sinus bradycardia, sinus arrest, no acceleration of sinus rhythm from effort, vasovagal syndromes, and others), can be treated with the use of implantable cardiac devices—pacemakers. 

The most commonly used definition of chronotropic failure is an inability to reach 85% of the maximum heart rate limit for one’s age during maximal exertion; however, it also encompasses delayed achievement of heart rate appropriate to the exercise intensity, inadequate variation in heart rate during exercise, or inadequate dynamics in returning to resting heart rate after exercise [3]. Disorders of automatism and conduction are characterized by the fact that they may come and go as conditions fluctuate, for example occurring with changes in body position or only at a particular heart rate [8]. The causes of CI are not fully understood. According to the available data, one possible cause is a decrease in the density and sensitivity of beta receptors, secondary to an increased sympathetic drive [9]. Undoubtedly, the use of beta-blockers, which are commonly used in patients with cardiac arrhythmias, has a strong influence on chronotropism [10,11,12]. CI is often observed in obese patients and in patients with type 2 diabetes, trisomy 21, rheumatoid arthritis, end-stage renal disease, kidney transplantation, or COVID-19 [13,14,15,16,17,18,19,20]. In patients with hepatic failure, the altered chronotropic response correlates with the degree of organ failure, making it a valuable criterion in cardiovascular assessment when selecting candidates for liver transplantation [21]. In patients that have undergone Fontan surgery, chronotropic failure correlates with impaired exercise capacity, liver dysfunction, and platelet abnormalities; the heart rate reserve in these patients may be an indicator of organ complications, and it may serve as a precursor for development of bradyarrhythmia and the future need for treatment with an implantable cardiac electronic device [22]. Within the population of pacemaker patients, predictors of CI include coronary artery disease, acquired heart valve disease, and the patient’s recorded condition after cardiac surgery. Interestingly, age and gender; left ventricular ejection fraction; time after pacemaker implantation; and the diagnosis of arterial hypertension, cardiomyopathy, or congenital heart defects have not been associated with the presence of CI in previous studies [23]. In terms of pharmacotherapy, the use of digitalis glycosides, beta-blockers, and amiodarone are important predictors of CI. The use of class I and IV antiarrhythmic drugs does not have a significant effect on CI risk [23]. Similar indicators have also been observed in studies of patients with an implanted cardioverter-defibrillator [24].

CI is a common problem in the heart failure patient population. According to the literature, it affects 25–70% of patients with heart failure; the wide range observed for this measure may result from the lack of a unified definition of CI and the use of different methods of assessment [25,26]. It is not clear whether the chronotropic failure is a cause or a consequence of the disturbed response of the organism to exercise in these patients [27]. In a group of patients with stable heart failure, partial improvement of physical capacity and reversal of chronotropic failure was achieved after cardiac rehabilitation using exercise training [28]. Comparing patients with pacemaker-dependent and non-pacemaker-dependent cardiac insufficiency, chronotropic and physical performance were worse in patients with pacemaker-dependent heart failure, which affected their quality of life and the effectiveness of cardiac rehabilitation. This issue, particularly, should be taken into account when selecting pharmacotherapy (especially beta-blockers; patients with a pacemaker that are also prescribed beta-blockers typically receive a higher average dose) and when making decisions about stimulation parameters influencing the heart rate during exercise [29].

## 3. Rate Response in Implantable Cardiac Pacemakers

### 3.1. Principles of Rate-Response Algorithms

A typical implantable cardiac pacemaker consists of a pulse generator and one, two, or three leads that are implanted, depending on the patient’s needs, endocavitary, into the muscularis of the right atrium, into the right ventricle, epicardially to the left ventricular wall, or directly or indirectly to the fibers of the conduction pathways or in their vicinity (stimulation of the bundle of His, stimulation of the left bundle branch area) [30,31,32]. Based on their construction, equipment, and technical capabilities, pacemakers are divided into classic subclavian-implanted (in most cases) systems, with leads inserted intravenously into the heart cavities, or leadless, endocavitary-implanted pacemakers [33]. The choice of type of pacemaker is made before implantation and is based on the indications for implantation, age, body structure (anatomical anomalies), comorbidities (diabetes, renal failure), and risk assessment of potential complications; additionally—in the case of patients previously treated with other cardiac implantable electronic devices—the presence of previous systems or components thereof (including abandoned or damaged leads), vascular patency in the planned course of electrode placement, and any history of past infectious complications will also be considered [34]. All currently produced pacemakers are equipped with a function to match the pacing frequency to the patient’s current metabolic demand (a rate-response function). However, the principles of operation; scope of application; methods of recording the variability of metabolic, hemodynamic, and physical parameters; and the method of processing signals related to this function can differ [35]. The general principle of the rate-response function is that it senses a specific factor that signals the start of physical activity or the existence of a situation related to increased oxygen demand. 

These pacemakers use built-in sensors to detect a signal that indicates the need for a faster heart rate and, in accordance with the programmed settings for the dynamics of the response and the permitted limit of the change in the pacing rate, calculates the number of sensor stimulations proportionally to the effort intensity; it increases the atrial (in pacing systems operating in single-chamber atrial-responsive pacing (AAIR mode) or dual-chamber sequential-responsive pacing (DDDR mode)) or ventricular (in single-chamber ventricular-responsive pacing (VVIR mode)) pacing rate to match the performed effort, up to the maximum pacing frequency limit defined by the parameter value (most often referred to as the "upper sensor rate") or until the effort ceases. In the parameters of the rate-response function, there is also a mechanism for setting the dynamics of a gradual return to the basic rate of pacing after the end of the exercise. This prevents sudden, significant drops in the stimulation frequency in situations where the effort is stopped abruptly. The parameters for the dynamics of increase and the dynamics of decrease, and for the limitation of the pacing rate, are programmable in order to individually adapt to the patient’s age, condition, needs, and lifestyle, taking into account their health history (it is also possible for multiple and noninvasive modifications to be made using the programming functions) [36,37].

Each type of sensor (categorized by the factor to which it responds) has its own specific capabilities, advantages, and limitations. A common solution involves two different sensors combined in one system in order to increase the sensitivity and specificity of stimulus perception by the system, optimizing the stimulation frequency change under the influence of the received factor and reducing the susceptibility to disturbances caused by interference from other stimuli acting on the sensed parameters [38]. 

According to the order in which they sense and react to the body’s increased demand for oxygen and the consequent need to increase the pacing frequency, the rate-response sensors can be divided into primary, secondary, and tertiary categories. The least physiological are the tertiary sensors, which detect parameters resulting from exercise, e.g., accelerometers. Secondary rate-response sensors detect parameters resulting from metabolic demand, e.g., minute ventilation. The most physiological sensors, termed primary sensors, detect parameters influencing cardiac function during exercise, e.g., closed-loop stimulation [39]. In terms of the sensed stimulus, the rate-response sensors can be divided into mechanical (accelerometer), electrical (impedance-based sensors), and algorithm-based (QT-interval, closed loop) categories. Table 1 presents selected technological solutions related to the rate-response functions in pacemakers currently available on the market, along with their characteristics.

### 3.2. Rate-Response Algorithms Based on Patient Activity

The simplest, most commonly used system for sensing a patient’s activity and the related need to adjust the pacing rate to their effort is an accelerometer that is built into the pacemaker’s pulse generator. In this type of sensor, the device detects the patient’s movement on the basis of the change in the position of the pulse generator, i.e., the deflection that naturally accompanies the movement of the chest while walking or running. The device continuously counts the number and frequency of recorded movements. It then converts this information into a proportional change in the pacing frequency in accordance with the dynamics programmed for a given patient, as well as the difference between the baseline pacing rate and the maximum limit to which stimulation may be increased during exercise. This sensor, however, has several significant disadvantages: it responds to increased effort with a delay and it is susceptible to disturbances in the form of external vibrations unrelated to, or disproportionately related to, the patient’s activity (e.g., while on a swing, riding over bumps, or riding a horse) [40,41].

**Figure 1:**
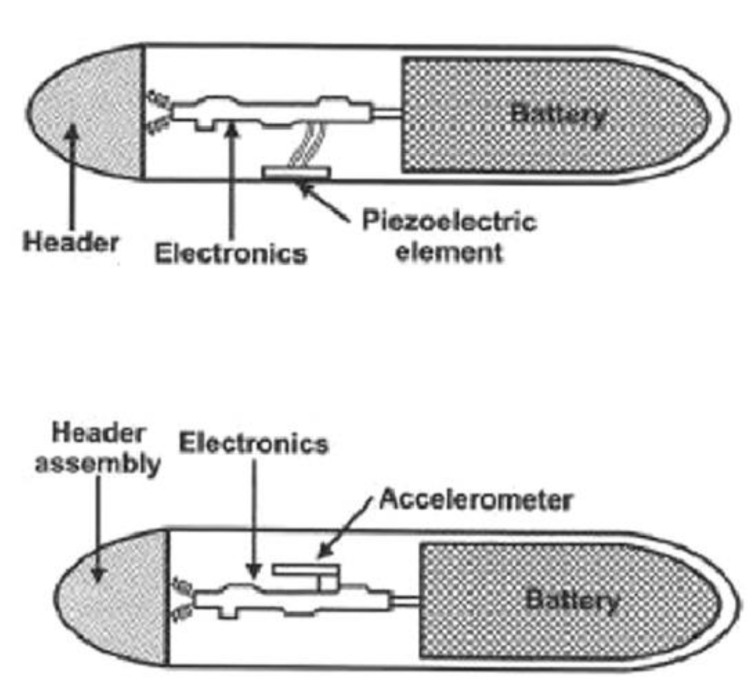
Scheme of the construction of a pulse generator with a built-in activity sensor based on an accelerometer and on a piezoelectric crystal. Adapted from reference [46] with permission.

The operation of an activity sensor based on the incorporation of a circuit with a piezoelectric crystal works similarly. In pacemakers with a rate-response function based on a built-in piezoelectric crystal, the sensed mechanical energy related to vibration, muscle tension, and body movement is converted into electrical signals, triggering an algorithm responsible for adjusting the pacing rate. Research studies have shown that sensors based on accelerometers more quickly and precisely select the frequency of stimulation appropriate to the effort than pacemakers with sensors based on the piezoelectric effect [40,42]. Despite this finding, a study that compared the primary endpoint of death or stroke and mortality, hospitalizations for heart failure, the incidence and duration of atrial fibrillation episodes, and the quality of life in patients with sinus node dysfunction (MOST Trial), found no statistically significant differences in patients with an accelerometer-based rate-response pacemaker when compared to patients with pacemakers with an embedded piezoelectric crystal. Interestingly, this study also observed a group of patients with blended-sensor pacemakers consisting of an accelerometer and piezoelectric circuit— this group of patients demonstrated significantly worse results in terms of quality of life and physical performance [43]. Figure 1 shows the construction of a pulse generator with a built-in accelerometer-based activity sensor and one with a piezoelectric crystal. Advanced work is being carried out on a new pacemaker rate-response system based on a piezoelectric sensor that has a higher sensitivity and accuracy in response to exercise. An additional, significant advantage is that the battery consumption due to system maintenance is decreased [44,45,46].

### 3.3. Rate-Response Algorithms Based on Minute Ventilation

**Figure 2:**
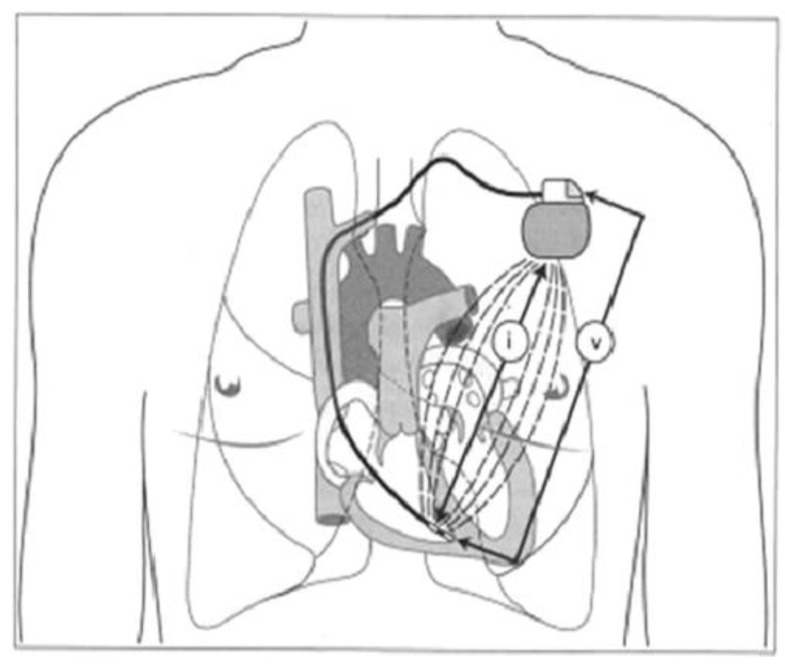
Diagram of the rate-response algorithm based on the assessment of minute ventilation by measuring intrathoracic impedance. Adapted from reference [46] with permission. Assessment of minute ventilation is based on constant measurement differences in voltage (v) between the tip of pacing lead and pacemaker can after masurement of current (i) between the ring of lead and pacemaker can.

Adjustment of the stimulation frequency can also be based on a minute-ventilation sensor, which acts as a physiological sensor of increased metabolic demand associated with stress or exercise. Minute ventilation, which is calculated on the basis of respiratory rate and tidal volume, correlates well with heart rate in patients without sinus node dysfunction; it may, therefore, serve as an ideal pattern to utilize for adjusting the pacing frequency to exercise in patients receiving cardiac pacing [47,48]. This system has also been successfully validated in pediatric patients [49,50]. Minute ventilation can be measured by assessing intrathoracic impedance due to fluctuating gas-to-tissue and -fluid ratios in the area throughout the respiratory cycle. In pacemakers, minute ventilation is based on monitoring changes in intrathoracic impedance measured in the circuit between the pulse generator and the tip of the pacing lead [51,52,53]. Figure 2 shows a diagram of the rate-response algorithm based on the assessment of minute ventilation by measuring intrathoracic impedance.

Unfortunately, this parameter has its limitations. It is susceptible to disturbances and changes in intrathoracic impedance that are not related to effort. Previously conducted measurements showed that the accuracy of this sensor may be impaired in situations of different body position (sitting vs. lying) and depending on the required effort (running vs. cycling) [54]. This sensor is also of limited use in patients with respiratory diseases [55]. A significant disadvantage of this solution is its susceptibility to interference associated with other medical equipment. There have been reports of inappropriate control of the pacing rate in patients with mechanical ventilation systems [56] and cardiac rhythm monitoring systems [57], and during transesophageal echocardiography [58]. There have also been reports of incorrect pacing frequency caused by sensor malfunction as a result of damage to the pacing lead [59,60]. In order to take advantage of the strengths and reduce the impact of the disadvantages of this system, a common solution is to combine a minute-ventilation sensor with an accelerometer-based sensor in a single pacemaker. This solution allows for the benefits of the physiological response of the minute-ventilation sensor (as opposed to the nonphysiological response of the accelerometer sensor), while also using a cross-check method that verifies the need to activate the rate-response algorithm using the observed movement recording by the accelerometer, reducing the risk of an inappropriate increase of the pacing rate in response to external disturbances [61,62]. 

Study results have shown that the use of this combination produces a greater improvement in heart rate scores compared to the use of an accelerometer alone; this is a measure that determines the risk of death in patients with an implanted cardioverter-defibrillator and allows for the identification of patients that may benefit more from rate-responsive stimulation [63]. The results for the use of two sensors are contradictory and inconclusive in terms of the impact on physical capacity and exercise tolerance for cardiac pacing, and thus this issue requires in-depth research to assess which patient types could benefit most from the use of combined responsive stimulation with two activity sensors [64].

### 3.4. Rate-Response Algorithms Based on the Analysis of the QT Interval

**Figure 3:**
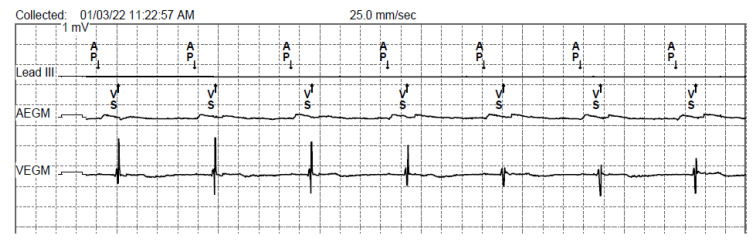
Example of an intracardiac EGM obtained from the recording leads of a dual-chamber pacemaker in bipolar mode. Speed of 25 mm/s. Voltage gain at 10 mm/mV. The recording shows a paced rhythm (atrial pacing, preserved A-V conduction, correctly recorded native ventricular beats). Markers "AP" indicates atrial paced events (peaks), markers "VS" indicates ventricular sensed events (beats).

Electrocardiographic QT-interval-based sensors provide a completely different solution for analyzing physiological increases in the body’s metabolic demand. These pacemakers continuously record the heart rhythm by calculating the potential difference between the poles of the pacing leads (bipolar pacing) or between the pole of the lead and the can of the pulse generator (unipolar pacing). This registration is called an intracardiac electrogram (EGM). An example of an intracardiac EGM obtained from the recording leads of a dual-chamber pacemaker in bipolar mode is shown in Figure 3.

Correct registration of intracardiac EGM signals forms the basis for the proper functioning of these pacemakers. Based on the EGM recording, the pacemaker calculates the time from the occurrence and sensing of a native or paced impulse. This allows it to send stimulation impulses at the appropriate time and control the heart rhythm according to programmed settings. The pacemaker also makes changes to basic pacing and sensing parameters using EGM. By automatically adjusting the sensitivity of the electrodes by adjusting the low- and high-pass filters, the pacemaker reduces the risk of incorrect pacing that could be caused by following incorrect signals registered by the lead (e.g., sensing muscle potentials, electromagnetic interference, incorrect classification of the QRS complex due to double or triple counting, incorrect classification of the T-wave or atrial fibrillation wave).

Based on the EGM recording, the pacemaker can also automatically assess the effectiveness of stimulation during periodic tests of the pacing threshold. In the absence of a deflection corresponding to the excitation of the atrium or ventricle after sending the test pacing impulses, the pacemaker can register ineffective stimulation. It can then change the amplitude of the stimulation output, reducing the risk of asystole. Additionally, the accurate recording of beats by the EGM allows for the use of algorithms that can change the atrioventricular interval to limit the pacing of the right ventricle. Excessive stimulation of the right ventricle (i.e., pacing in a situation where atrioventricular conduction is preserved with a delay that does not affect the deterioration of hemodynamic parameters) increases the risk of developing poststimulating cardiomyopathy [65]. 

In the majority of currently used cardiac implantable electronic devices, it is possible to automatically manage the pacing of the right ventricle. This is achieved by gradually extending the atrioventricular (A-V) interval in order to evaluate whether a native ventricular beat can be detected after a sensed or paced atrial beat [66].

QT interval analysis is a tool for the management of pacing rate during exercise (rate response) based on electrographic recordings (EGM) [67,68]. This algorithm is based on analysis of the duration of changes in the QT interval during exercise and rest after sending a single unipolar impulse during ventricular depolarization [69]. Under exercise or stress conditions, myocardial contractility increases in response to autonomic nervous system regulation, resulting in a change in the duration of the QT interval corresponding to the period of ventricular repolarization [70,71,72]. The atrioventricular interval, depending on the native atrioventricular conduction time or the programmed A-V interval settings on the pacemaker, is also closely related to the QT interval [73,74,75,76]. Recent studies describe the phenomenon of QT adaptation as an abrupt change in the rate or interruption of atrial and ventricular pacing; this is indicative of the complexity of the phenomenon and the existence of many potential factors (extrinsic, such as pacing, and intrinsic, such as pharmacotherapy of conduction dysfunctions, electrolyte disturbances, or short- or long-QT syndromes) that can affect this parameter [77,78,79,80,81]. One of the disadvantages of this sensor type is the slow response to effort. Numerous attempts have been made to improve response time and to allow diversification of the rhythm and pacing frequency distribution to achieve a nearly physiological distribution of heart rate in a human undertaking various forms and degrees of intensity of exercise [82,83,84,85].

Unfortunately, pacing rate control based on the analysis of changes in the QT interval has been associated with a susceptibility to malfunction and inadequate acceleration or deceleration of pacing under nonexercise conditions affecting QT interval duration. Additionally, inappropriate control of rate modulation pacing has been reported in association with drugs that affect the duration of myocardial repolarization [86]. In patients with coronary artery disease, there is a particular need for care when using QT-based rate modulation pacing settings. In these patients, ischemic pain may induce a pacemaker response in the form of an increase in heart rate by increasing the adrenergic response, which carries a small risk of increasing pain in a vicious circle by increasing the heart rate [87,88,89]. 

In patients with an implanted pacemaker that modulates the pacing rate based on QT interval analysis, changes in the pacing rate have been observed both during and after fever [90]. Due to the ability to monitor subtle changes in the QT interval and the response to physiological changes associated with inflammatory processes, an attempt was made to use this method as a noninvasive tool for diagnosing early signs of rejection in heart transplant recipients; however, the trials did not result in a sufficient level of diagnostic sensitivity and specificity to allow the solution to be considered clinically useful [91]. As with the previously described sensors, the combination of two different methods for recognizing increased metabolic demand could eliminate the impact of the disadvantages and enhance the advantages of using algorithms based on the analysis of the QT interval. Currently used pacemakers are, however, not equipped for this type of monitoring of physical activity [35,92].

### 3.5. Rate-Response Algorithms Based on Closed-Loop Stimulation

**Figure 4:**
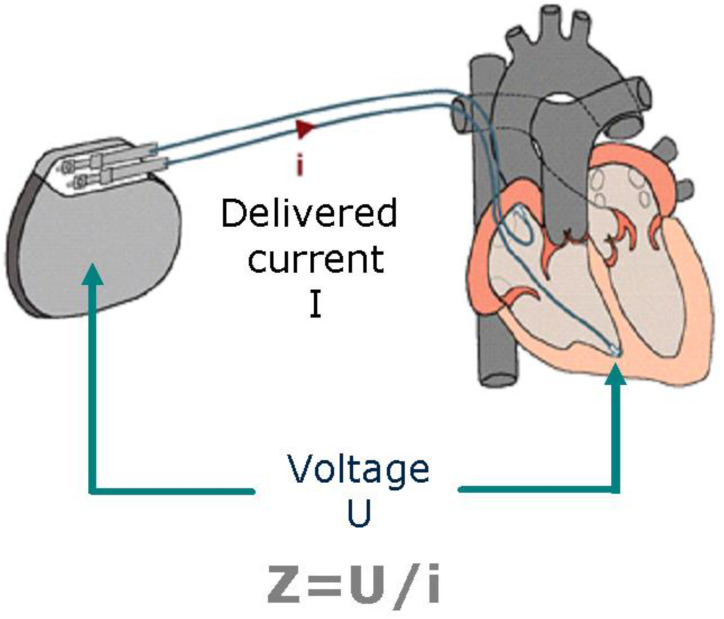
Diagram of a functioning CLS pacemaker. Reproduced with permission of BIOTRONIK, Berlin, Germany.

An innovative type of rate-response sensor is based on the closed-loop stimulation (CLS) algorithm. The principle behind this method is the continuous observation (in a closed loop) of changes in unipolar intracardiac impedance related to the filling of the ventricles with blood in the diastolic phase and the change in the ratio of the volume of fluid (blood) to tissue (walls of the myocardial chambers) in the contraction phase, under the direct influence of stimuli from the autonomic nervous system [93]. Figure 4 shows a diagram of a functioning CLS-based rate-response pacemaker.

**Figure 5:**
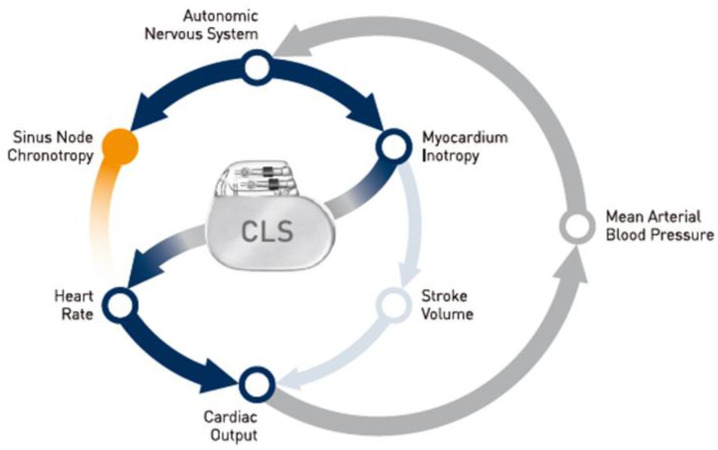
Scheme of the closed-loop stimulation algorithm. Reproduced with permission of BIOTRONIK, Berlin, Germany.

In CLS systems, a series of subthreshold electrical pulses are sent to enable impedance measurements in the tissue immediately surrounding the tip of the pacing lead placed in the chamber. A pacemaker with a CLS system adjusts the pacing rate by continuously analyzing the rate of change in impedance associated with changes in myocardial contractility during increased metabolic demand caused by emotions, exercise, or other conditions. It then calculates the difference between gradient sums of actual and reference impedance measurements [94]. CLS sensors are one of the most physiological options among the currently used cardiac implantable devices because they detect and respond to parameters influencing cardiac function simultaneously with the changing demand, rather than secondarily after the recording of parameters resulting from exercise or metabolic demand. This ensures a proportionate improvement in physical capacity and allows daily living activities to be performed with less effort [95,96,97]. Figure 5 shows a diagram of the function of this algorithm. 

**Figure 6:**
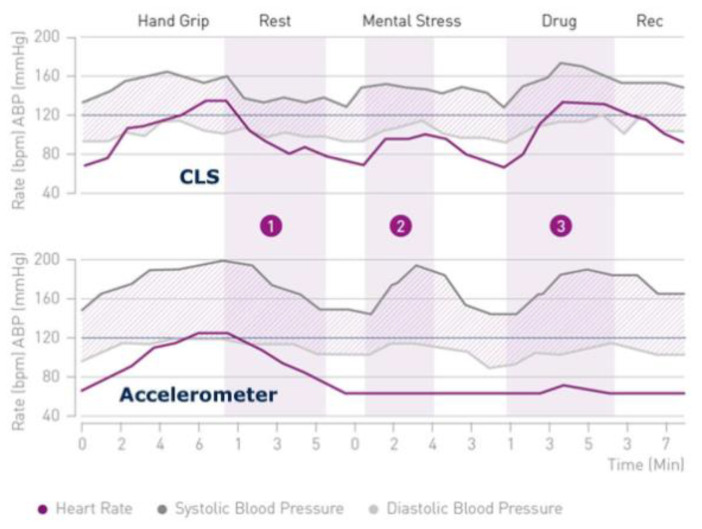
Scheme of the changes in the impedance of the right ventricle during the heart cycle in closed-loop stimulation and accelerometer pacing. Reproduced from Reference [100].

CLS (Biotronik, Germany) works based on the measurement of intracardiac impedance of the area of about 0.5–1 cm diameter around the ventricular electrode (blood and endocardial tissue), on the basis of which it builds a model of the instantaneous work of the heart (details shown in Figure 6). Changes in the impedance of the right ventricle are correlated with dP/dtmax of the right ventricle, which corresponds to the rate of contraction of the right ventricle. Thus, during the contraction of the heart, there is greater contact between the electrode and the endocardial tissue, which increases the impedance and, as a result, sends information about the need to increase the heart rate—i.e., the stronger the contraction, the greater the impedance causing the acceleration of the heart rate. Changes in intracardiac impedance are often illustrated by impedance curves, and changing the shape of these curves causes CLS activation. Despite the known mechanism of action of CLS and the undeniable benefits in counteracting vasovagal syndrome (VVS) syncope, it is not fully known what activates this algorithm during such an episode—it may be the acceleration of the activity and the increase in the force of contraction of the heart in the first phase of the presyncope state, which is a physiological response to prevent it. Activation of CLS in this mechanism also allows the minute volume to be maintained at the time of pressure drop during the vascular reflex. After the reflex (when CLS-activating factors have subsided), the cardiac pacing rate gradually returns to baseline values according to the pacing program. It should be mentioned that there are no universal recommendations for programming CLS in the case of VVS, and the program of the device should be set individually so that the patient does not feel discomfort due to the increased frequency of stimulation or its overlong duration. It seems that in cases of VVS, it may be beneficial to program a higher CLS activation [98,99,100,101].

Due to the principle underlying the CLS sensor, it characteristically reduces symptoms associated with syncope in patients with vasovagal syndrome. Several clinical trials have evaluated the use of CLS in patients with vasovagal reflex syncope, with a mean follow-up of 30 months, and 88% of patients experienced a reduction in symptoms associated with vasovagal syndrome after using CLS [102,103,104,105].

In accelerometer-equipped pacemakers, the rate-drop response (RDR) is used to prevent vasovagal syncope. This function is based on the short period of overstimulation after an episode of sudden decrease in heart rate is detected (cardiodepressive mechanism of vasovagal syncope).

The main disadvantage of this function is the late reaction. The rate-drop response is activated after the beginning of the vasovagal mechanism, which often starts with a hypotensive reaction. The pacemaker detects a decrease in heart rate, which can precede syncope as a result of hypotension. Consequently, patients with vasovagal syncope achieve only partial improvement in the form of reducing the number of syncopes and prolonging the prodromal period, allowing them to adopt a safer body position before the syncope.

Furthermore, the rate-drop response function is based on a nonphysiological, sudden increase in the pacing rate, which is sometimes not well tolerated by patients, interpreted as long-lasting (even up a minute) attacks of palpitations which accompany the feeling of upcoming syncope [106]. Closed-loop stimulation is based on continuous analysis of intracardiac impedance, which changes along with the heart’s work cycle and under the influence of mechanisms that change myocardial contractility. 

In clinical trials comparing the effectiveness of CLS and RDR in preventing vasovagal syncope, superior efficacy of CLS devices has been evidenced [102]. This effect was also observed in patients with an epicardial lead [107]. Consequently, in the latest European Society of Cardiology (ESC) 2021 pacing guidelines, the implantation of a CLS pacemaker in patients >40 years of age with severe, recurrent, unpredictable reflex syncope caused by asystole was placed in class I indications (recommended) [34,108].

Moreover, this sensor is capable of detecting and responding to fluctuating demands of cardiac output due to blood flow and electrolyte changes associated with hemodialysis renal replacement therapy [109]. In a retrospective analysis of patients diagnosed with paroxysmal atrial fibrillation with an implanted pacemaker with a closed-loop sensor, a lower burden of atrial fibrillation was observed when compared to patients with a pacemaker without rate response or with a different sensor [110,111].

The limitation of this method is the need to implant a right ventricular lead (an algorithm impossible to use in AAIR systems). Furthermore, some patients experience accelerated pacing that is unsuitable for their needs and individual tolerance range. Additionally, the use of negative inotropic drugs and the presence of postinfarction scars in the area of the implanted right ventricular lead may reduce the effectiveness of the algorithm [35]. Currently, this algorithm is used only in implantable cardiac devices from BIOTRONIK, Germany.

### 3.6. Rate-Response Algorithms Based on Peak Endocardial Acceleration

Another solution for using an accelerometer as the basis for a rate-response function is to optimize the pacing rate in response to isovolumic contraction (and the first cardiac tone) and isovolumic relaxation (and the second cardiac tone) intervals. Technically, this is performed by analyzing endocardial vibration sensed using an accelerometer built into the tip of the ventricular lead (usually ventricular, but it is also used in systems with an accelerometer lead placed in the atrium). This solution allows for the assessment of systolic isovolumic peak acceleration—mechanical activity of the heart which increases during adrenergic stimulation, a parameter of heart contractility. It can also be used to follow the changes in heart rate and demand during exercise. The disadvantage of this solution is the need to use a dedicated electrode compatible with the pulse generator (Sorin Group, Saluggia, Italy). This prevents this solution from being used in patients in whom there are no indications for replacement or additional implantation of the leads from previously used systems, even when replacing the pulse generator itself due to battery depletion [112,113,114,115].

### 3.7. Rate-Response Algorithms Based on Transvalvular Impedance (TVI)

An example of pacing rate modulation based on hemodynamic parameter analysis is the rate-response function based on measurements of and changes in transvalvular impedance (TVI). TVI is measured via electrical impulses in a circuit created between the ends of the atrial and right ventricular leads. This method is based on the fact that changes in the impedance of this area are associated with changes in the filling of the heart chambers with blood during the heart cycle. The TVI reaches a minimum value during atrial systole (which corresponds to the end-diastolic phase of the cycle) and a maximum at the end of the QT period (which corresponds to the end-systolic phase) [116,117]. The maximum TVI corresponds to the end-systolic volume and is sensitive to changes in cardiac contractility, which allows this solution to be used to express the autonomic nervous system’s regulation of the heart in patients with chronotropic insufficiency [118].

Changes in the TVI waveform correspond to echocardiographic recordings of filling parameters used in the evaluation of myocardial contractility under the influence of right ventricular stimulation. This allows for an indirect assessment of changes in ventricular contractility with stimulation from the septal region compared to the apical region of the right ventricle [119]. Diagnostic information on trends in TVI, due to reliable correlation with hemodynamic parameters in preclinical studies, may prove valuable in cardiac disease therapy in patients with an implanted device [120]. This system is available in Medico (MEDICO S.R.L., Rubano, Italy) cardiac devices.

### 3.8. Rate-Response Optimization Parameters

Each manufacturer uses different technological solutions in the process of translating information about registered movement into an adjustment in pacing rate. The possible setups of pacemakers produced by different manufacturers are presented below. The information is the individual opinions of the authors, based on commonly available materials, mainly product manuals, and our own interpretations supported by clinical experience. The statements are not authorized by medical equipment manufacturers and may only be used by medical personnel under their own responsibility [100,121,122,123,124,125].

**Figure 7:**
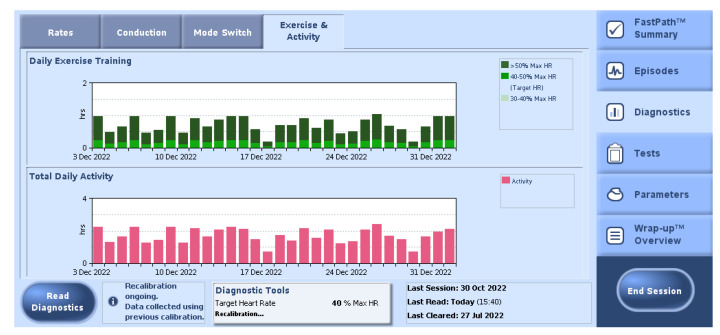
Example of physical activity statistics recorded by a dual–chamber pacemaker (Abbott, Chicago, IL, USA).

In the most commonly used devices, the rate-response sensor setup can be programmed as on, off, or passive. In passive mode, rate-response sensors do not adjust pacing parameters to patient movement, but only register the activities (in range of time spent on activity and exertion intensity expressed as percent of heart rate maximum) for diagnostic purposes (Figure 7 shows example of this registration). 

The main modifiable parameters of the rate-response function are threshold, slope, reactive time, and recovery time.

The threshold parameter is responsible for recognizing whether activity occurred. In the case of a pacemaker that under- or overreacts to patient activity, the threshold can be adjusted by changing the default sensitivity, where it is possible to set the result of automatic measurements (e.g., auto +1 or −1, etc.) or set the parameter fixed on one constant value. Setting a threshold value over the automatic assessment makes the pacemaker less sensitive to movement (i.e., a greater effort will be necessary to recognize activity by the pacemaker and as a result to adjust pacing parameters). Proper setup of threshold parameters prevents unnecessary overreaction of the pacemaker during slight movements of the body caused by factors other than physical activity (including muscle tremors that occur with neurological disorders).

**Figure 8:**
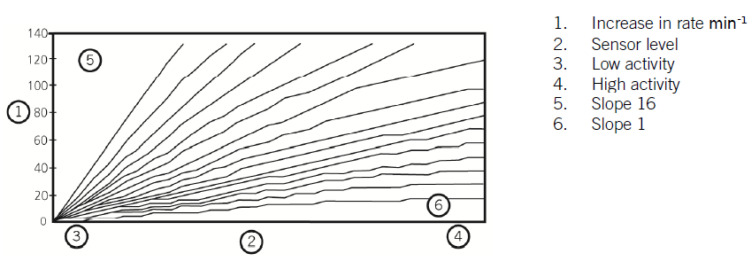
Scheme of slopes illustrating increases of pacing rate (Abbott, Chicago, IL, USA). Reproduced from Reference [121].

The slope (or response factor) is responsible for determining the change in pacing rate when the threshold recognizes activity. Slope defines the proportion of pacing rate change, taking into account the intensity of effort and the difference between the lower rate and max sensor rate, linearly dividing this difference for 16 degrees of intensity (Abbott, St. Jude Medical, Boston Scientific). Similar to the threshold parameter, the slope can be programmed in automatic mode, which can be adjusted (e.g., +1, −0.5) or set as a fixed value in range of 1–16. Figure 8 shows the scheme of these slopes based on the Abbott (IL, USA) devices.

Reaction time is responsible for mitigation of the change from a lower pacing rate to a rate assessed by the pacemaker as adequate to ongoing activity. The purpose of this function is to prevent abrupt changes in the pacing rate. The reaction time parameter can be set in different modes, e.g., slow/medium/fast/very fast. 

Recovery time is responsible for mitigation of the change of pacing rate from the highest stage of activity to a lower rate after the effort has ended. Recovery time can be set in modes, e.g., very slow/slow/medium/fast.

Apart from the lower rate/base rate and max sensor rate/upper sensor rate, in Medtronic/Vitatron devices, another parameter is applied to define rate-response pacing: the ADL rate (activities of daily living rate). This parameter defines the target desired pacing rate during light/moderate activities of daily living (walking, household activities). 

**Figure 9:**
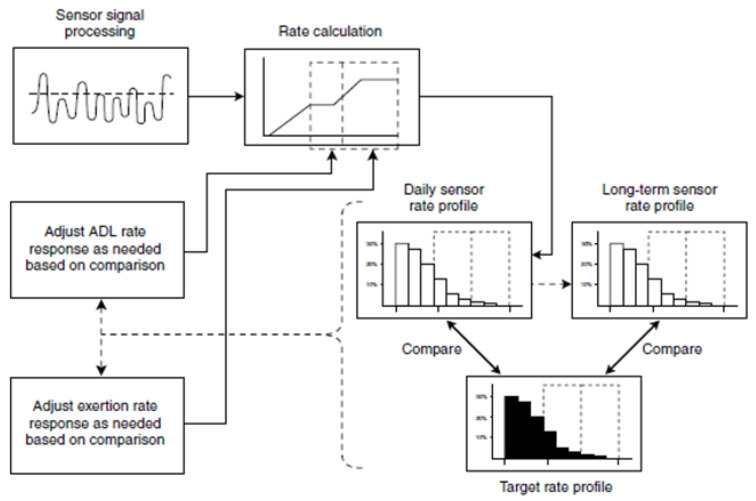
Scheme of Rate Profile Optimization (Medtronic, Minneapolis, MN, USA). Reproduced from Reference [122].

In devices manufactured by Medtronic/Vitatron, the function of Rate Profile Optimization is available. This algorithm, based on registered physical activities, adjusts the increase of pacing rate in the first stages of physical effort as well as during training with volatile intensity in the ADL rate range and exertion rate range. Figure 9 shows the scheme of this algorithm.

In devices commonly used in Europe, blended rate-response sensors are available, for example accelerometer and minute-ventilation (MV) sensors. This kind of pacemaker is described below, based on devices manufactured by Boston Scientific. In these devices, both sensors (or one of them) can be programmed to be active or inactive simultaneously.

During mechanical ventilation of a patient, deactivation of the MV sensor is indicated. In patients with respiratory disorders or other states in which risk of incorrect functioning of MV sensors is higher, individual assessment of deactivation or modification parameters should be considered (see details in Section 3.3., “Rate-response algorithms based on minute ventilation”).

The principle of the minute-ventilation sensor is the analysis of the difference between short-term and long-term transthoracic impedance statistics.

These statistics are continuously collected, updated, and analyzed. Long-term statistics are updated every 4 min and short-term statistics every 7.5 s, which allows for a quick reaction to the changing physiological conditions that accompany sudden physical effort. For patient comfort and safety, the increase or decrease in heart rate caused by activation of the rate-response function is limited to a maximum of 2 bpm in each cardiac cycle.

**Figure 10:**
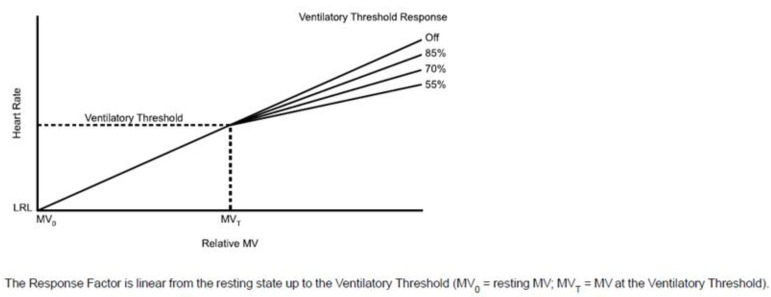
Scheme of ventilatory threshold response (Boston Scientific, Marlborough, MA, USA). Ventilatory threshold response is responsible for adapting the pacing rate in the range between the ventilatory threshold value and the max sensor rate value, expressed in %, which corresponds to the deflection of the curve determining the intensity of pacing rate adaptation. Reproduced from Reference [123].

The ventilatory threshold parameter is intended to reflect the physiological point beyond which ventilation increases faster than VO2. In pacemaker algorithms, this value, expressed in beats per minute, can be programmed manually or automatically calculated by the device based on the patient’s gender and age (if they have been entered into the device’s memory in the appropriate way) and the patient’s lifestyle, determined by the fitness level parameter in terms of sedentary/active/athletic/endurance sports (details in Figure 10).

A graphical representation of the patient’s activity and sensor responses ("Sensor trending graph") is helpful in optimizing the device for the activity of the rate-response function parameters.

For patients with both sensors active, the pacemaker averages calculations of the rate estimated as appropriate by each of these sensors separately. In a situation where the rate indicated by the accelerometer is lower than that indicated by the minute-ventilation sensor, then the pacemaker will pace 100% according to the indications from the minute-ventilation sensor.

If the accelerometer indications are higher than those calculated from minute ventilation, the pacemaker will take into account both sensors, changing the dominance of these sensors depending on the heart rate indicated by the accelerometer (from lower rate to max sensor rate), starting at approximately 80% for the accelerometer decision, 20% for MV sensor decision if accelerometer rate is at the lower rate limit, reaching 40% for accelerometer and 60% for minute ventilation if the rate estimated by the accelerometer is at the max sensor rate.

## 4. Accelerometer as a Solution for Maintenance of Atrioventricular Synchrony in a Leadless Pacemaker

Leadless pacemakers are an alternative to classic pacemakers, with leads placed intravenously in the heart cavities. This solution is especially recommended for patients in whom implantation of a classic pacemaker is impossible or difficult for various reasons (e.g., as a result of anatomical anomalies or obstacles in the form of inactive or damaged leads from previously used pacing systems, patients experiencing complications in therapy with implantable cardiac devices) and in patients at a particularly increased risk of infectious complications (including infective endocarditis) related to the presence of a foreign body in the cardiovascular system [126,127].

This risk is increased in patients with a prior history of infections related to cardiac implantable electronic devices (device pocket infections, endocarditis, lead-infective vegetations, and systemic infections due to the presence of an implanted pacemaker) and in patients with systemic diseases, diabetes mellitus, or kidney failure [128]. The leadless pacemaker (Micra ^®^, Medtronic, MN, USA) consists of a small, capsule-shaped generator with hooks at the end that allow fixation of the pacemaker in the wall of the right ventricle. Inside the pulse generator, in addition to the battery and the computer system that controls the pacemaker’s algorithms, there is an accelerometer that allows the pacemaker to properly control the rate of stimulated impulses [129].

In the single-chamber leadless system, working in the VVIR mode, the accelerometer provides the signals for the rate-response algorithm. The system reacts by accelerating the stimulation rate when it detects an increased frequency and intensity of vibration related to the patient’s physical effort and adjusting the pacing within the set parameters, i.e., according to the dynamics of the increase and decrease of the stimulation frequency and according to the set maximum frequency [130]. This system is most commonly used in patients with persistent atrial fibrillation with a slow ventricular rate.

**Figure 11:**
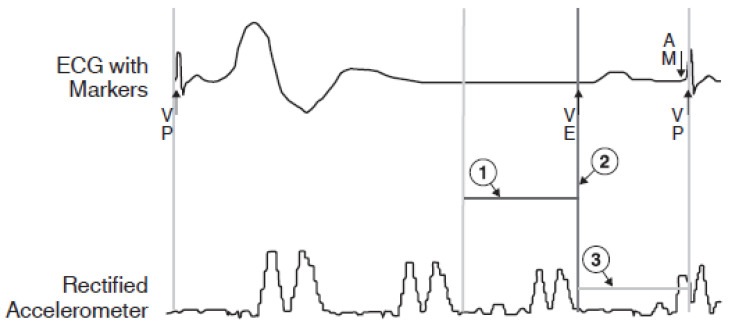
Diagram of the heart rhythm recorded by a leadless pacemaker. 1. A3 threshold value allows for proper sensing without interference. 2. End of A3 window (diastole). 3. A4 threshold line allows for proper sensing of atrial systole. Reproduced from Reference 133 with permission from Medtronic, Minneapolis, MN, USA.

**Figure 12:**
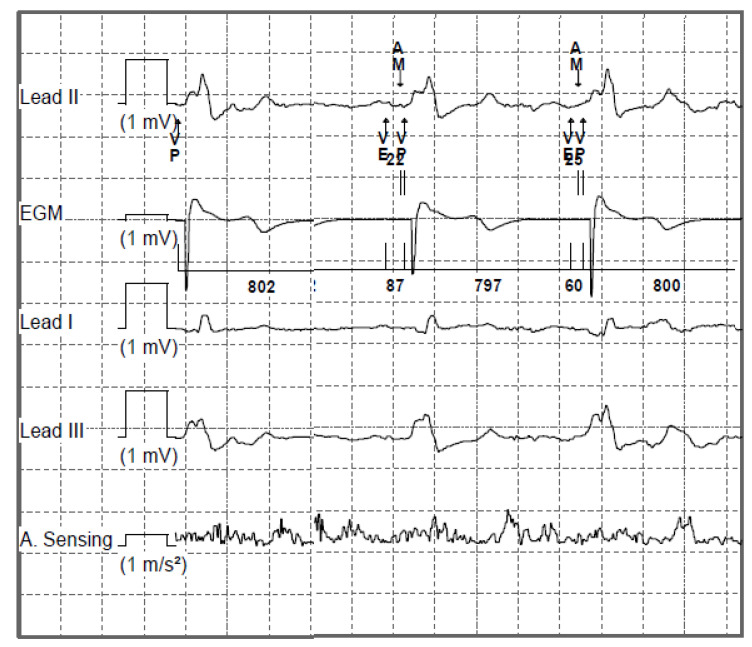
Example of an intracardiac EGM obtained from the recording of a leadless pacemaker in VDD mode. Speed of 25 mm/s. Voltage gain at 10 mm/mV. The lowest channel shows atrial activation sensed by the accelerometer built into the pacemaker, expressed in m/s^2^.

The highly advanced version of the leadless pacemaker is a device adapted to sense and control pacing under the influence of sensed atrial activity (VDD mode pacing). In leadless VDD pacemakers (Micra AV ^®^, Medtronic, MN, USA), the accelerometer, in addition to the typical function of sensing increased metabolic demand in situations of physical exertion, senses the mechanical wave associated with blood flow through the valves after atrial contraction. The registration of the mechanical wave accompanying the outflow of blood after atrial systole, during the opening of the mitral and tricuspid valves, allows for the appropriate timing of ventricular pacing in a patient with impaired atrioventricular conduction. This solution allows ventricular pacing to be controlled by the intrinsic function of the atria without physically inserting the lead into the right atrial cavity. Figure 11 shows a scheme of heart rhythm recording in leadless VDD systems. Figure 12 shows an example of this registration. The limitation of this solution is the inability to perform atrial pacing, which excludes patients with sinus node dysfunction from treatment with this type of pacemaker [131,132,133].

## 5. Discussion

The main role of an implanted pacemaker is to support the work of the heart in disorders such as sinus node insufficiency or conduction blocks. Proper pacemaker programming—in accordance with the individual needs resulting from the type of arrhythmia, the indications for pacemaker implantation, and the patient’s lifestyle and preferences—has a crucial impact on improving heart function. It is also important to avoid replacing the intrinsic rhythm with pacing unnecessarily, due to the risk of pacing-induced cardiomyopathy [134].

Modern implantable cardiac electronic devices are equipped with numerous algorithms, the task of which is to optimize the pacing settings to match the physiological function of the heart as closely as possible. It is difficult to imagine the heart functioning without being able to adapt the heart rate to situations related to physical effort of various intensities or stress levels. The technological solutions used for recording and processing information based on the real-time demand and volatility of minute capacity differ. This creates the opportunity to select an appropriate device, equipped with the functionalities that offer the greatest potential benefit to the patient. The daily experience of working with patients with cardiac implantable electronic devices, combined with many years of case observation, has revealed no shortage of evidence or examples of diametrical changes in well-being following pacing parameter adjustments in terms of rate-response settings. This is especially true in patients with a high percentage of atrial pacing. However, with regard to the impact of the rate-response function on the change in patients’ physical capacity, the data are not clear; there are only a few results from contemporary studies available, though further studies exploring this issue are in progress [135].

There is evidence to support the use of rate-responsive pacing in patients with permanent atrial fibrillation and VVIR pacing systems [136]. However, in patients receiving dual-chamber sequential pacing, studies have not been able to conclusively demonstrate the benefit of frequency-adaptation stimulation [43,137].

The lack of unequivocal results from clinical trials on this subject may be due to differences in the effectiveness of the adaptation of the stimulation frequency resulting from technological differences in the types of sensors described in this article, as well as differences in the way they are used. This includes the parameters that are programmed related to the function, such as maximum pacing values during exercise and the settings for the dynamics of the increase and decrease of the pacing rate. The method used to calculate the maximum pacing frequency limit appropriate for a given patient may also be important. A study conducted from 2017 to 2019 showed that programming the rate-response function in patients with heart failure with reduced left ventricular ejection fraction under the control of echocardiography improved the duration of physical effort; this was in contrast to the most commonly used programming of this function depending on the patient’s age, which, in the study’s opinion and based on their results, may cause deterioration of cardiac systolic function and the worsening of heart failure in patients with CI and heart failure [138,139,140].

Currently, the most commonly used method for evaluating the distribution of heart rate in a patient with a cardiac-implanted device, when assessing the applied pacing parameters, is the analysis of the heart rate histograms stored in the memory of the pacemaker. These are based on the recorded intervals of successive heartbeats on the intracardiac EGM [141]. Based on the intracardiac EGM signal, some implantable devices automatically optimize settings (to a limited extent) for rate response and rate smoothing [142].

## 6. Summary

In summary, the use of the pacemaker rate-response function is a promising tool that can potentially improve the quality of life and physical capacity of patients, but this tool requires additional research efforts and further clinical trials; this will allow for a better understanding of the mechanisms responsible for the effect of this therapy. Additional data are needed to broaden and popularize knowledge relating to the complexities of the technical aspects underlying the process of optimizing the stimulation parameters, giving medical personnel the opportunity to take full advantage of the option to program this function.

## Figures and Tables

**Table 1:** Technological solutions related to rate-response functionality and their characteristics.

Type of Sensor	Construction of the Sensor	Method of Sensing and Interpreting Signals	The Trigger of the Reaction	Advantages	Disadvantages
Activity-based accelerometer	Accelerometer built into pacemaker pulse generator	Movement (swings) detected by counting the “peaks” on the accelerometer to adjust stimulation rate	Movement of body	The most common, simple sensor, applicable in a majority of devices	High sensitivity to interference, nonphysiological and unproportional response to exercise, undersensitivity to certain types of activity, insensitive to emotional stress
Activity-based piezoelectric crystal	Piezoelectric crystal built into pacemaker pulse generator	Vibrations sensed by the piezoelectric crystal, translated into electrical signals to adjust stimulation rate	Movement of chest	A common, simple sensor, low consumption of the pacemaker battery	High sensitivity to interference, nonphysiological and unproportional response to exercise, undersensitivity to certain types of activity, insensitive to emotional stress
Minute ventilation	Circuit between pulse generator and pacemaker lead	Measures of changes in thoracic impedance related to physical effort	Respiratory rate, gas volume in the lungs	Physiological sensor connected with the real oxygen demand in exercise, responsive to mental and emotional stress	Limited specificity of reaction in patients with respiratory disorders, underreaction in the initial phase of exertion, high risk of interference with other medical devices
QT-interval-based sensor	Computed algorithm for analysis of QT interval	QT interval detected by leads and analyzed by device	Change of QT interval associated with physical, mental, or emotional stress	Responsive to all kinds of exercise and stress	May be underreactive or hyperreactive in some patients, especially those with a long QT syndrome or arrhythmogenic diseases, or patients treated with QT-prolonging medications
Closed-loop impedance-based contractility sensor (BIOTRONIK, Germany)	Measurement of impedance tissue around the tip of the ventricular lead	Measures of changes in intracardiac impedance associated with contractility	Increases in contractility of heart muscle	Responsive to all kinds of exercise and stress, additional effect of prevention of vasovagal syncope	Undersensitive for patients after myocardial infarction or patients taking negative inotrope drugs
Activity-based contractility sensor (PEA sensor) (Sorin Group, Italy)	Accelerometer built into tip of pacemaker’s lead	Measures of maximum endocardial acceleration	Detection of first and second cardiac tone related to isovolumic contraction and relaxation	Responsive to all kinds of exercise and stress, precise measurement related to hemodynamic parameters	Necessity of using a dedicated lead compatible with the pulse generator
Transvalvular impedance (MEDICO S.R.L., Italy)	Circuit between the atrial and ventricular lead	Hemodynamic response for exercise	Changes of transvalvular impedance which increases during ventricular systole and decreases during the passive and active filling period.	Responsive to all kinds of exercise and stress, precise measurement related to hemodynamic parameters	Available only in dual-chamber pacing systems

## Data Availability

Not applicable.
